# From benign appearance to malignant truth: a case report of mesenteric dedifferentiated liposarcoma with inflammatory myofibroblastic tumor-like features

**DOI:** 10.1186/s13000-025-01640-3

**Published:** 2025-04-10

**Authors:** Simon Schallenberg, Miriam Schulte, Mihnea P. Dragomir, Armin Jarosch, Wolfgang Hartmann, Eva Wardelmann

**Affiliations:** 1 Institute of Pathology, Charité-Universitätsmedizin Berlin, Corporate Member of Freie Universität Berlin and Humboldt-Universität Zu Berlin, Berlin, Germany; 2 Gerhard-Domagk-Institut Für Pathologie, Universitätsklinikum Münster, Münster, Germany; 3 Partner Site Berlin, and German Cancer Research Center (DKFZ), German Cancer Consortium (DKTK), Heidelberg, Germany; 4 Berlin Institute of Health, Berlin, Germany

## Abstract

Dedifferentiated liposarcoma (DDLPS) with inflammatory myofibroblastic tumor (IMT)-like features is a rare and diagnostically challenging variant of soft tissue sarcoma. We report the case of a 74-year-old man who presented with a mesenteric mass in 2022 and recurrent tumors in 2024. Tissue from both primary and recurrent tumors were submitted to our reference center for pathological reevaluation, with a suspicion of IMT being suspected. Although the tumors exhibited morphological characteristics consistent with those observed in IMT, they displayed distinctive histological, immunohistochemical and molecular features suggestive of DDLPS with IMT-like features, including amplification of the *MDM2* gene. This report highlights the morphological spectrum of DDLPS, the diagnostic role of molecular pathology, and the importance of differentiating this aggressive neoplasm from benign entities such as IMT.

## Introduction

Dedifferentiated liposarcoma (DDLPS) is an atypical lipomatous tumor/well-differentiated liposarcoma (ALT/WDLPS) that progresses, either in the primary or in a recurrence, to a non-lipogenic sarcoma of variable histological grade. It represents approximately 10–20% of all liposarcomas [1–3]. It has been established that approximately 90% of cases arise de novo, while 10% develop from recurrences [1–3]. DDLPS is most often found in the retroperitoneum but can also occur in the spermatic cord and more rarely in head and neck, mediastinum, trunk or mesentery [1, 4–10].

The World Health Organization (WHO) classification of soft tissue tumors delineates five main subtypes of liposarcoma: well-differentiated, dedifferentiated, myxoid, pleomorphic, and myxoid pleomorphic liposarcomas. The histological hallmark of DDLPS is the transition from the WDLPS component to a higher-grade, non-lipogenic sarcomatous component [1, 2]. Morphologically, the dedifferentiated areas may exhibit a broad histological spectrum, most frequently resembling undifferentiated pleomorphic sarcoma or leiomyosarcoma and, in rare cases, inflammatory myofibroblastic tumor (IMT)-like features [11–16].

IMT is a low-grade spindle cell neoplasm typically associated with anaplastic lymphoma kinase (*ALK*) and more rarely with ROS oncoprotein (*ROS1*) or neurotrophic tropomyosin kinase receptor (*NTRK*) rearrangements and a less aggressive clinical course compared to DDLPS [17–19]. IMT-like DDLPS variants, however, pose a significant diagnostic challenge due to overlapping histological features, such as myxoid, cellular, and hypocellular fibrous stroma, chronic inflammatory infiltrates as well as areas closely resembling fibromatosis or nodular fasciitis [11, 12]. Misdiagnosis of IMT-like DDLPS as a benign entity such as IMT, desmoid-type fibromatosis, and reactive myofibroblastic lesions can lead to suboptimal treatment strategies [11, 12].

Molecular pathology plays a pivotal role in resolving these diagnostic dilemmas. The amplification of *MDM2*, by definition detected in DDLPS, serves as a hallmark molecular signature [20]. Conversely, the absence of *ALK, ROS1* or *NTRK* rearrangements and of *CTNNB1* mutations help to exclude IMT and desmoid fibromatosis, respectively.

In this report, we present the case of a 74-year-old male with a rare mesenteric DDLPS exhibiting IMT-like features, diagnosed initially in 2022 and presenting with recurrent tumors in 2024. This case underscores the importance of integrating histopathological, immunohistochemical, and molecular findings to navigate the complex differential diagnosis of mesenchymal tumors. Additionally, we aim to provide a review of the literature, highlighting diagnostic and therapeutic implications for this rare variant of DDLPS.

## Case description

A 74-year-old male patient was admitted in 2022 with a primary mesenteric mass. In 2024, three additional subserosal tumors were identified in the mesentery, with the largest measuring 5 cm. Subsequently, all tumors were excised via surgical resection. Tissues from all tumors were submitted to our reference center for pathological reevaluation, with an external diagnosis of IMT being suspected.

## Histological examination and molecular pathology

**Fig. 1:**
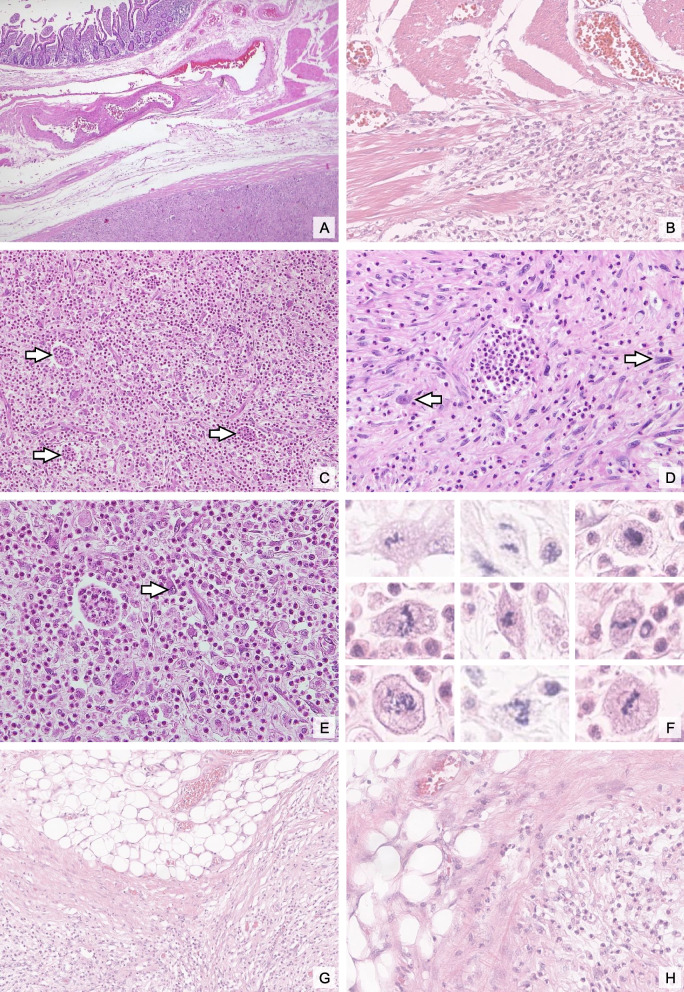
Histomorphological characteristics: Intestinal wall cross-section with the enteral mucosa visible in the top left and DDLPS with infiltration of the tunica muscularis propria visible at the bottom right (**A**). Higher magnification highlighting tumor cell infiltration of the tunica muscularis propria (**B**). The lower magnification shows pronounced inflammatory infiltrates, characterized by a predominance of eosinophils and neutrophils and the formation of microabscesses (white arrows; **C**). In the tumor areas with lower inflammatory cell density, the bizarre tumor cells with pleomorphic nuclei (white arrows) are clearly visible (**D**), while in tumor areas with pronounced inflammatory infiltrates (**E**), even in the higher magnification, the tumor cells are not clearly visible (white arrow). There was a significant increase in the number of mitoses (**F**). No adjacent atypical lipogenic tumor component was found (G+H); hematoxylin-eosin, original magnifications x12.5(A), x40 (**C**, **G**) and x200 (**B**, **D**, **E**, **H**) and 400x (**F**)

The histological examination of the primary tumor revealed the presence of a centripetal infiltration of the intestinal wall, extending to the tunica muscularis propria (Fig. 1A + B). At low magnification, both primary and recurrent tumors displayed a myxoid and loosened stroma, interspersed with dense inflammatory infiltrates predominantly composed of eosinophils and neutrophils (Fig. 1C). These inflammatory cells formed microabscesses in several regions of the tumor, contributing to its distinctive appearance. At higher magnification, single bizarre tumor cells were evident, characterized by pronounced pleomorphism and hyperchromatic nuclei, indicative of malignancy (Figs. 1D and E). A marked increase in mitotic activity was observed, with up to 7 mitotic figures per 10 HPF (Fig. 1F). No necrosis was identified. The tissue surrounding the tumor consisted of morphologically unremarkable adipose tissue. No adjacent atypical lipogenic tumor component was present; however, MDM2 FISH was not performed on that component for clarification (Fig. 1G + H).

**Fig. 2:**
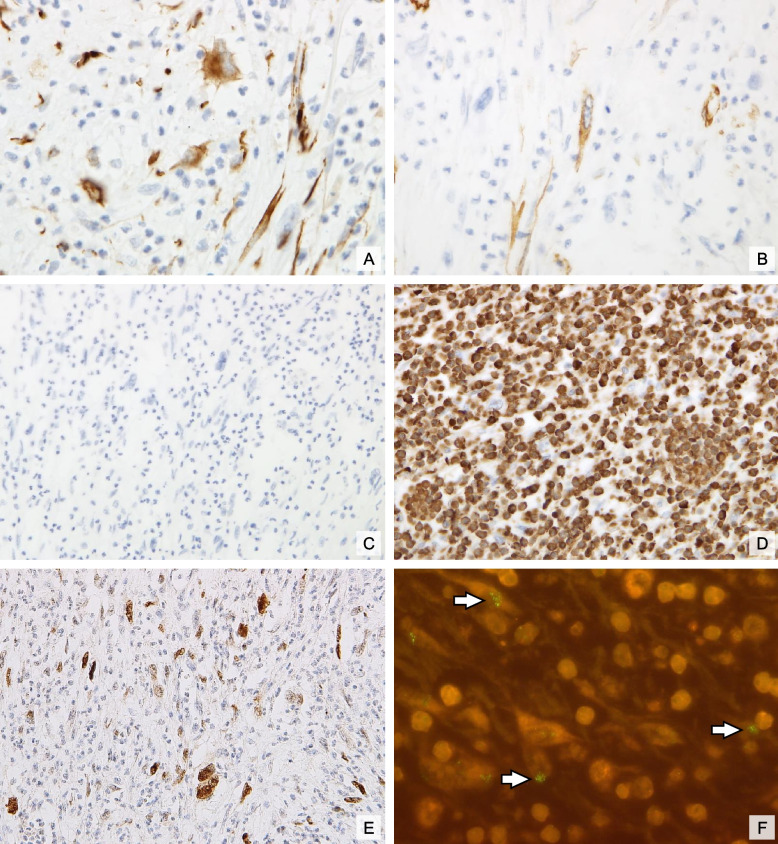
Immunohistochemical and molecular features: Immunohistochemical reactivity for desmin (**A**) and smooth muscle actin (**B**) confirms myogenic differentiation, while the ALK staining was negative (**C**). CD15 marks the pronounced granulocytic infiltrate (**D**). Nuclear positivity for MDM2 and *MDM2* amplification via FISH (*MDM2* clusters are indicated by white arrows) confirms the diagnosis of DDLPS (**E**, **F**); original magnifications × 400 (A,B), × 200 (**C**-**E**) and × 600 (**F**)

Immunohistochemically, individual tumor cells showed cytoplasmic expression of desmin and smooth muscle actin (SMA), indicating a partial myogenic differentiation (Figs. 2A and B). The tumor cells were negative for pan-Trk, ROS1, and anaplastic lymphoma kinase (ALK; Fig. 2C). Heterogeneous expression of p53 was noted, reflecting *TP53* wildtype phenotype. The proliferation index, determined by Ki-67 staining, reached an average value of 20%, illustrating the high proliferative activity of the tumor cells. Immunohistochemical staining revealed strong nuclear expression of MDM2 (Fig. 2E).

Further confirmation through fluorescence in situ hybridization (FISH) showed high-level amplification of the *MDM2* gene, evident as clustered signals (Fig. 2F). This amplification of *MDM2*, combined with the other histological and immunohistochemical findings, conclusively established the diagnosis of DDLPS with IMT-like features, underscoring the tumor’s aggressive biological behavior and diagnostic complexity. According to the classification system of the French Fédération Nationale des Centres de Lutte Contre le Cancer (FNCLCC), the tumor was classified as grade 2 (G2).

## Discussion

This case highlights the diagnostic and clinical challenges posed by DDLPS with IMT-like features, emphasizing the need for a multimodal diagnostic approach. The mesenteric location of this DDLPS adds another layer of rarity, as DDLPS most commonly occurs in the retroperitoneum or extremities [1, 4–9]. The dedifferentiated component in this case exhibited histological features closely mimicking IMT, including myxoid stroma and dense inflammatory infiltrates. This pattern is consistent with the IMT-like variant of DDLPS described in the literature [11, 12]. Other histological patterns of DDLPS have been observed, including myxofibrosarcoma-like, leiomyogenic, osteosarcomatous, chondrosarcomatous, angiosarcomatous, rhabdomyosarcomatous, neural-like or meningothelial-like whorling patterns [1, 5, 21–23]. The diagnostic challenge lies in distinguishing IMT-like DDLPS from other entities. Although the histology is similar, in IMT, frequently ALK, ROS1 or pan-Trk expression can be demonstrated by immunohistochemistry as indicator of an underlying *ALK*, *ROS1* or *NTRK* gene rearrangement [18]. Desmoid fibromatosis typically exhibits bland fibroblastic spindle cells in a collagen-rich stroma, lacks a dense inflammatory infiltrate and shows immunohistochemical nuclear ß-catenin expression. Tumors cells lack nuclear atypia and *MDM2* amplification as seen in DDLPS. The detection of *MDM2* cluster amplification as in this case is a hallmark of DDLPS, aiding in its distinction from benign and low-grade mimickers, such as atypical spindle cell/pleomorphic lipomatous tumor or solitary fibrous tumor. Performing *MDM2*-FISH analysis on a mesenchymal spindle cell tumor in the abdomen or the retroperitoneum is essential for the definitive exclusion of liposarcoma. However, this molecular finding does not assist in distinguishing DDLPS from ALT/WDLPS, as both sarcomas exhibit amplification of *MDM2* and most often *CDK4*. While morphological differentiation is crucial in this context, it is often challenging, particularly in the sclerosing and inflammatory subtypes. Sclerosing ALT/WDLPS is characterized by scattered bizarre stromal cells with hyperchromatic nuclei within a dense collagenous stroma, while inflammatory ALT/WDLPS features chronic inflammatory infiltrates and bizarre multinucleated stromal cells [24–26]. A key feature distinguishing ALT/WDLPS from DDLPS is the presence of mature adipocytes, that typically predominate in ALT/WDLPS but are found only focally in DDLPS [23]. Conversely, DDLPS presents with a dedifferentiated, typically non-lipogenic, low or high-grade morphology that may even overgrow any residual lipogenic features [1, 2]. Furthermore, Harry Evans proposed a mitotic count of ≥ 5 mitoses per 10 HPF as a valid criterion for differentiating DDLPS from ALT/WDLPS, which aligns with our observation of up to 7 mitotic figures per 10 HPF [4, 27].

DDLPS is an aggressive tumor with a high rate of local recurrence and a significant risk of distant metastasis. It is graded as G2 or G3 according to the FNCLCC grading system [28]. Furthermore, myogenic differentiation may be associated with a worse outcome [28]. In the presented case, recurrent tumors developed within two years of initial diagnosis in 2022, consistent with the unfavourable reported clinical behavior of mesenteric DDLPS [11]. Complete surgical resection remains the fundamental treatment approach. Nevertheless, achieving negative margins in mesenteric regions is frequently challenging due to the complex anatomy, substantial tumor burden, and infiltration of surrounding structures and organs [12, 29–31]. The available evidence indicates that radiation therapy may potentially reduce the risk of local recurrence in DDLPS. Nevertheless, the effect of this treatment on overall survival remains unclear [32–34]. The role of adjunctive chemotherapy is still a topic of debate within the scientific community [31, 32, 35, 36].

The prognosis for DDLPS is variable and dependent on a number of factors, including tumor grade, location (with retroperitoneal lesions exhibiting the poorest clinical outcome) and resectability [28]. Five-year survival rates range between 20–40% [29, 30]. The IMT-like variant does not appear to alter the overall prognosis significantly but complicates the diagnostic process, increasing the risk of misdiagnosis and delayed treatment [15, 37].

## Conclusion

In conclusion, the IMT-like variant of DDLPS represents a diagnostic pitfall that requires careful histopathological and molecular evaluation. This case underscores the importance of an integrated diagnostic approach and highlights the need for continued research into this aggressive tumor subtype.

## Data Availability

No datasets were generated or analysed during the current study.
